# Relationship of Abdominal Circumference and Trunk Length With Spinal Anesthesia Block Height in Geriatric Patients Undergoing Transurethral Resection of Prostate

**DOI:** 10.7759/cureus.33476

**Published:** 2023-01-07

**Authors:** Muhammad Yahya, Aliya Ahmed, Iayla Fatima, Muhammad Nasir

**Affiliations:** 1 Anaesthesiology, Aga Khan University Hospital, Karachi, PAK; 2 General Surgery, St. Luke’s General Hospital, Kilkenny, IRL; 3 Anaesthesiology, South City Hospital, Karachi, PAK

**Keywords:** transurethral resection of prostate, block height, abdominal circumference, trunk length, turp, spinal anaesthesia

## Abstract

Introduction: Spinal anesthesia is commonly used for various surgical procedures. Prediction of spinal anesthesia block height is always a challenging task for anesthetists. Higher than desired levels of spinal anesthesia blocks are associated with serious side effects, while inadequate block height does not provide satisfactory surgical anesthesia. In this study, we observed the relationship between the ratio of trunk length (TL) and square of the abdominal circumference (AC^2^) and spinal anesthesia sensory block height in geriatric patients undergoing transurethral resection of the prostate (TURP).

Material & Methods: This is a cross-sectional study conducted at the Aga Khan University Hospital Karachi, Pakistan, on geriatric patients undergoing TURP under spinal anesthesia. Forty-three elderly patients (American Society of Anaesthesiology level I-III) between 60 and 80 years were recruited for the study. In hospital wards, trunk length (TL) and abdominal circumference were recorded before the procedure. In the operating rooms, spinal anesthesia was performed at L3-L4 intervertebral space with 0.5% hyperbaric bupivacaine 10mg (2mls). Block height was measured by the placement of ice pads at different dermatomes. Spearman rank correlation coefficient was used to analyze the physical parameters (TL/AC^2^) and spinal anesthesia block height.

Results: The ratio of trunk length and square of the abdominal circumference (TL/AC^2^) correlates with spinal anesthesia block height in geriatric patients, where the spearman rank correlation coefficient was r =-0.284 with p = 0.015.

Conclusion: The ratio of the long axis (TL) and transection area of the abdomen (AC^2^), which coincides with (TL/AC^2^), correlated with spinal anesthesia sensory block height. Hence, elderly patients with a low TL/AC^2 ^ratio will have higher block height after spinal anesthesia.

## Introduction

Spinal anesthesia is commonly used for various surgical procedures in obstetrics, orthopedics, gynecology, and urology. It is well known that spinal anesthesia is associated with procedure-related complications, including post-dural puncture headache (PDPH) (0.16%-1.3%) [1], transient neurological symptoms (10.2%-24%) [2], spinal epidural hematoma (1:2,700-1:190,000) [3], and excessive block height (0.2%) [4].

Excessive block height is associated with a higher incidence of adverse effects of spinal anesthesia, such as nausea, vomiting, bradycardia, and hypotension [5]. However, the inadequate spread of local anesthetics does not provide satisfactory surgical conditions [6]. Therefore, it is important to be able to predict and control the intrathecal spread of local anesthetics and spinal anesthesia block height (SABH).

Factors that affect SABH have been investigated in multiple studies, and contributing factors include clinical technique used, characteristics of drug solution injected, and patients’ general features, i.e., age, height, weight, and sex [7,8]. In the geriatric population, there is a small but significant increase in SABH, rate of onset of motor block, and cardiovascular instability regardless of the drug solution used [9-13]. These changes are linked to age-related changes in spinal anatomy, nerve physiology, and cardiovascular reflexes [7]. Increased intra-abdominal pressure, changes in anteroposterior spinal curves, and lumber lordosis are also associated with increased SABH [14-17]. However, measuring these parameters is difficult in daily practice.

Hartwell et al. found that vertebral column length had a much better correlation than body height to predict the spread of subarachnoid hyperbaric bupivacaine in term parturient [18]. Lee and co-researchers found in 30 terms Taiwanese parturient that Trunk Length (TL)/Abdominal Circumference^2^ (AC^2^) values, which simulated the ratio of the long axis and transection area of the abdomen, correlate with maximal SABH, and parturient with low TL/AC^2^ values tended to have high SABH with standardized spinal anesthesia [8].

The study mentioned above showed clinically significant results in term parturient. The parameter TL/AC^2^ was statistically correlated with high SABH (Spearman correlation coefﬁcient, -0.45 with p < 0.02) [8]. However, no such relationship was investigated in geriatric patients shown to have variation in SABH compared to younger adults [9-13]. Geriatric patients often have multiple co-morbidities, and side effects or complications of spinal anesthesia are bound to be more troublesome for these patients [19]. Therefore, to avoid complications or side effects, it is important to be able to predict and control SABH more precisely and accurately in geriatric patients. This study has improved our understanding of the relationship of the SABH with TL/AC^2^ in the geriatric patient population.

## Materials and methods

This cross-sectional study was conducted in the operating rooms of Aga Khan University Hospital in Karachi, Pakistan, from 20^th^ December 2016 to 19^th^ June 2017. The sample size was calculated based on previous research in which the correlation coefficient between the ratio of TL/AC2 and SABH was -0.45 (p-value 0.02) [8]. For a spearman correlation coefficient of at least 0.45, which is the minimum value to have clinical interest, and the probability of type I error ≤ 0.05, the sample size must be at least 29 for 80% power and 40 for 90% power. To increase power, we included 45 patients in this study. A non-probability consecutive sampling technique was applied. All adult male (American Society of Anaesthesiology level I-III) patients, aged 60 to 80, undergoing Trans-Uretheral Resection of Prostate (TURP) electively under spinal anesthesia, were included in the study. On the other hand, exclusion criteria included obesity (BMI > 30 kg/m^2^) [20,21], cachexia (BMI < 20kg/m^2^) [22], patient refusal, known spinal abnormalities (kyphosis, scoliosis, lordosis), history of spinal surgeries, large abdominal mass (in the past or present), tumor or ascites, and height less than 150 cm and more than 170 cm.

Data collection procedure

After approval from the ethical review committee of Aga Khan University, written informed consent was obtained from all the participants. Before coming to the operating room, measurements (TL/AC^2^) of all the patients fulfilling the inclusion criteria were taken in the ward. Abdominal circumference was measured (in cm) in the supine position with a measuring tape at the level of the umbilicus at end-expiration, and trunk length (in cm) in the left lateral decubitus position from C7 spinous process to sacral hiatus. After starting intravenous fluids (0.9% normal saline) and establishing routine peri-operative monitoring (i.e., non-invasive blood pressure, pulse oximeter, three lead ECG), spinal anesthesia was administered with an aseptic technique using a 25-gauge pencil-point spinal needle wherein 0.5% hyperbaric bupivacaine (10 mg) was injected at L3-L4 spinal level after confirmation of free flow of cerebrospinal fluid (CSF) in sitting position. Patients were put in a supine position immediately after the successful spinal injection. Sensory block height was measured by the application of an ice pad over different dermatomes every five minutes after the successful spinal injection for 20 minutes. Surgery was allowed to start after the achievement of the T-10 level block.

Data analysis procedure

All statistical analyses were performed using IBM Corp. Released 2010. IBM SPSS Statistics for Windows, Version 19.0. Armonk, NY: IBM Corp. Mean and standard deviation (SD) were computed for age, height, weight, trunk length, abdominal circumference, and the ratio of TL/(AC)^2^. Frequency and percentage were calculated for ASA level. Spearman correlation (non-normal data) between the ratio of TL/(AC)^2^ and maximum block height (spinal level) was computed, and p≤0.05 was considered significant. Stratification analysis was performed to observe the confounding effect of AC and BMI on the correlation between TL/(AC)^2^ and maximum block height (spinal level).

Operational definitions

Trunk Length in cm: In left lateral decubitus position from C-7 spinous process to sacral hiatus with measuring tape [8]. C-7 spinous process: The largest and most inferior spinous process in the neck. It can be identified with the palpation/flexion/extension method [23]. Sacral hiatus: The opening into the vertebral canal in the midline of the dorsal surface of the sacrum. It can be identified by palpation of sacral cornua at the lower end of the sacrum. Abdominal Circumference in cm: In supine position at the level of umbilicus with measuring tape. Sensory block: Unable to appreciate cold sensations when ice cube placed over the anesthetized area. Block Height: Unable to appreciate cold sensations at the level of Umbilicus = T-10, Xiphoid process = T-6, Nipple = T-4. L3-L4. Level identification: The line joining the superior aspect of the iliac crests posteriorly (the intercristal line) is L4-L5, and the interspace is just above L4-L5. Geriatric age: Chronological age of 60 years and above [24].

## Results

**Figure 1:**
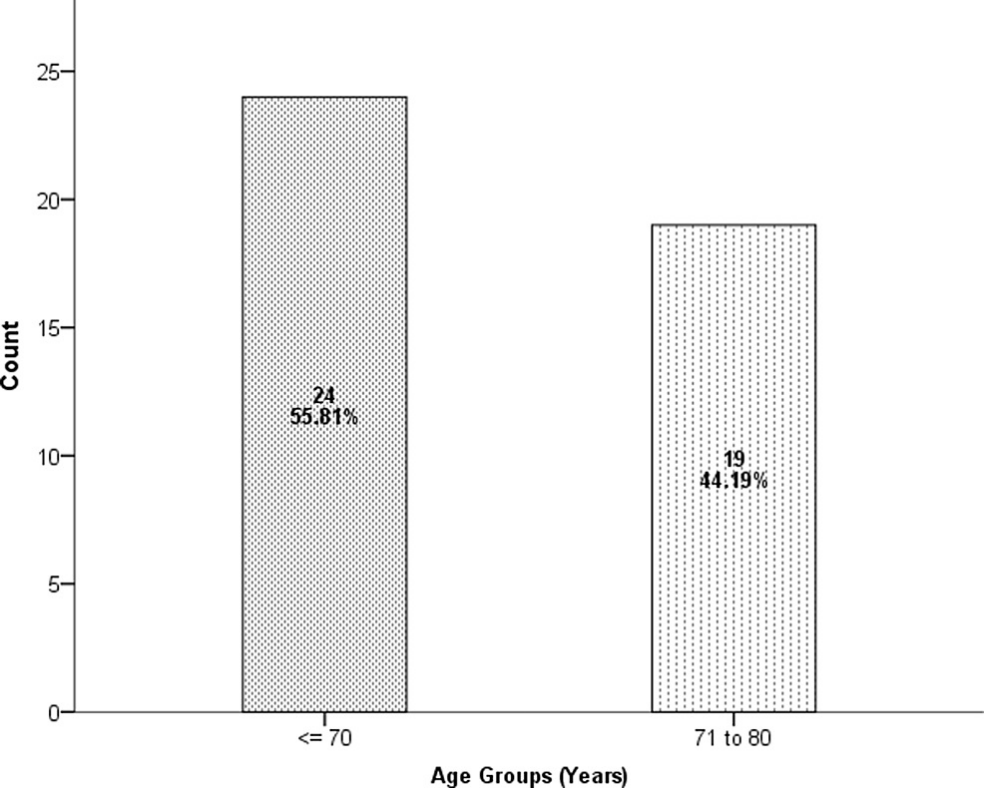
Age Distribution of the Patients

A total of 45 geriatric patients, scheduled to undergo TURP under spinal anesthesia, and fulfilling inclusion criteria, were enrolled for the study. Two of them refused, perioperatively, repeated measurements of sensory block height with ice pad placement and hence were excluded from the study. The rest of the 43 patients were included in the study. Out of 43 patients, 24 were between 60 and 70 years, and 19 were between 70 and 80 years (Figure 1).

**Figure 2:**
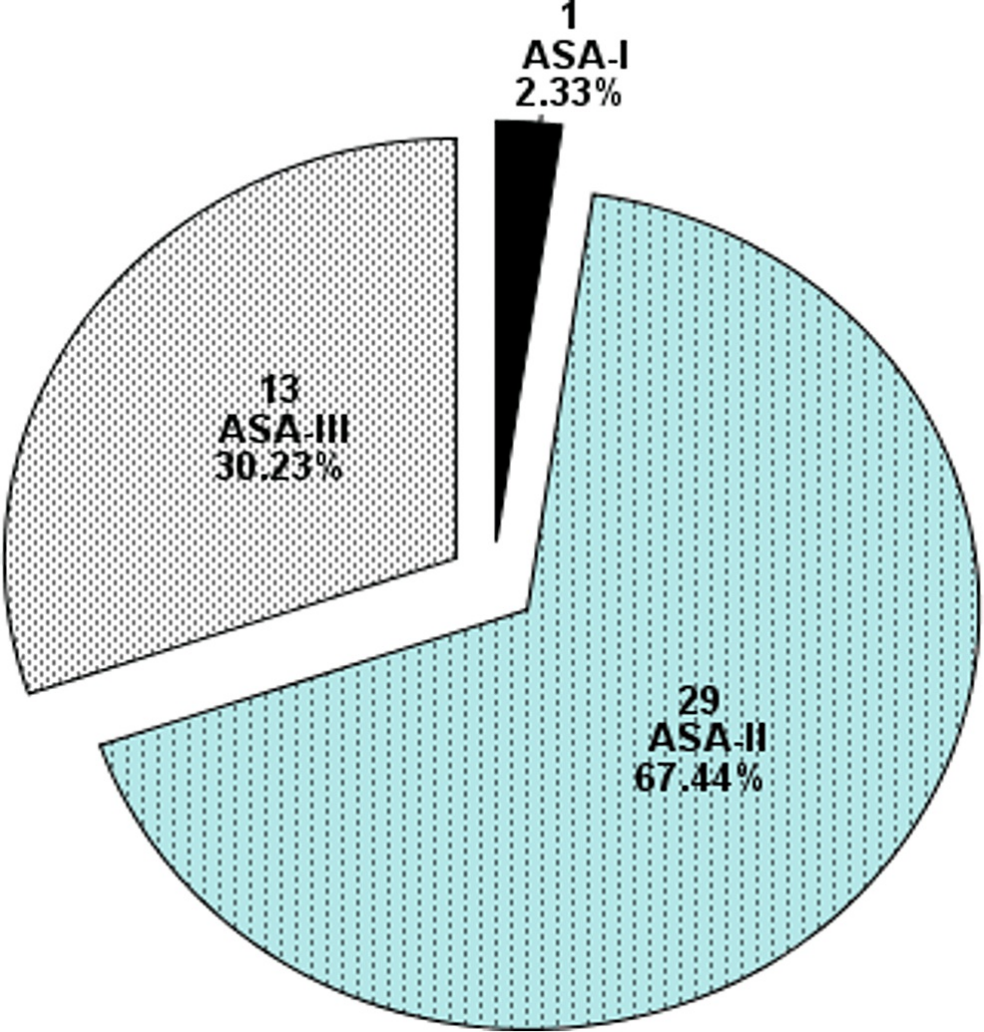
ASA Distribution of the Patients

However, the age of the patients was 69 +/-6.47 (Mean +/- SD) years, as shown in Table 1. Other demographic variables are also shown in Table 1. Regarding ASA status, 2.33% were ASA-I, 67.44% were ASA-II, and 30.23% were ASA-III, as shown in Figure 2.

**Table 1:** Demographic Characteristics of Patients n= 43

Variables	Mean	SD	95% Confidence Interval for Mean		Min	Max
			LowerBound	UpperBound		
Age (Years)	69	6.47	67.01	70.99	60	80
Weight (kg)	71.08	10.73	67.78	74.38	50	90
Height (cm)	164.84	5.48	163.15	166.52	150	170
BMI (kg/m^2^)	26.21	3.09	25.25	27.16	20.02	31.14

**Figure 3:**
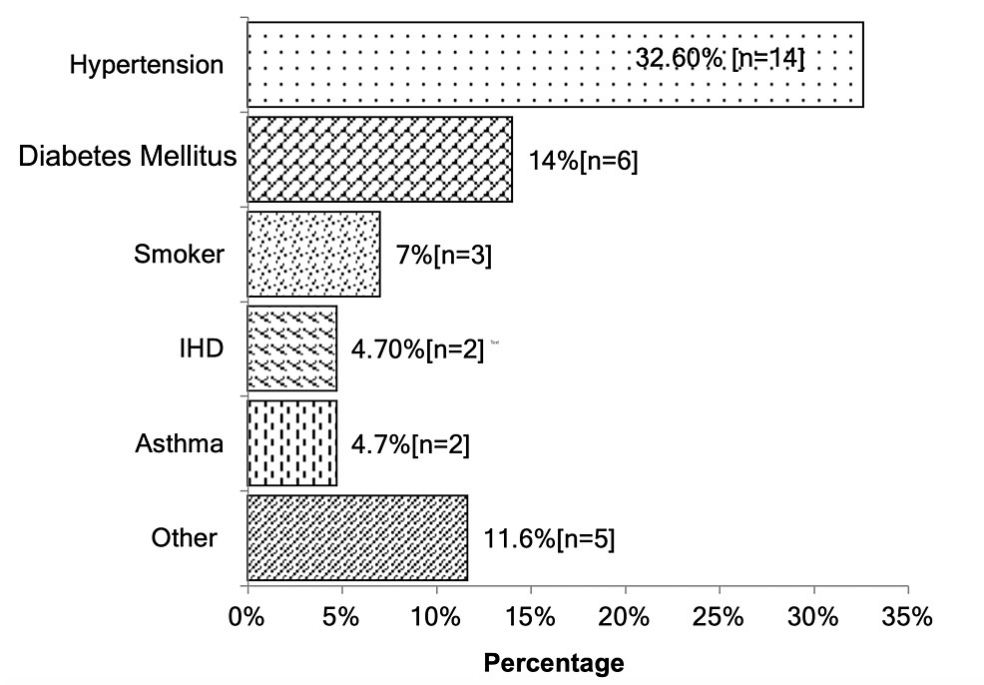
Co-morbidities of Patients

**Figure 4:**
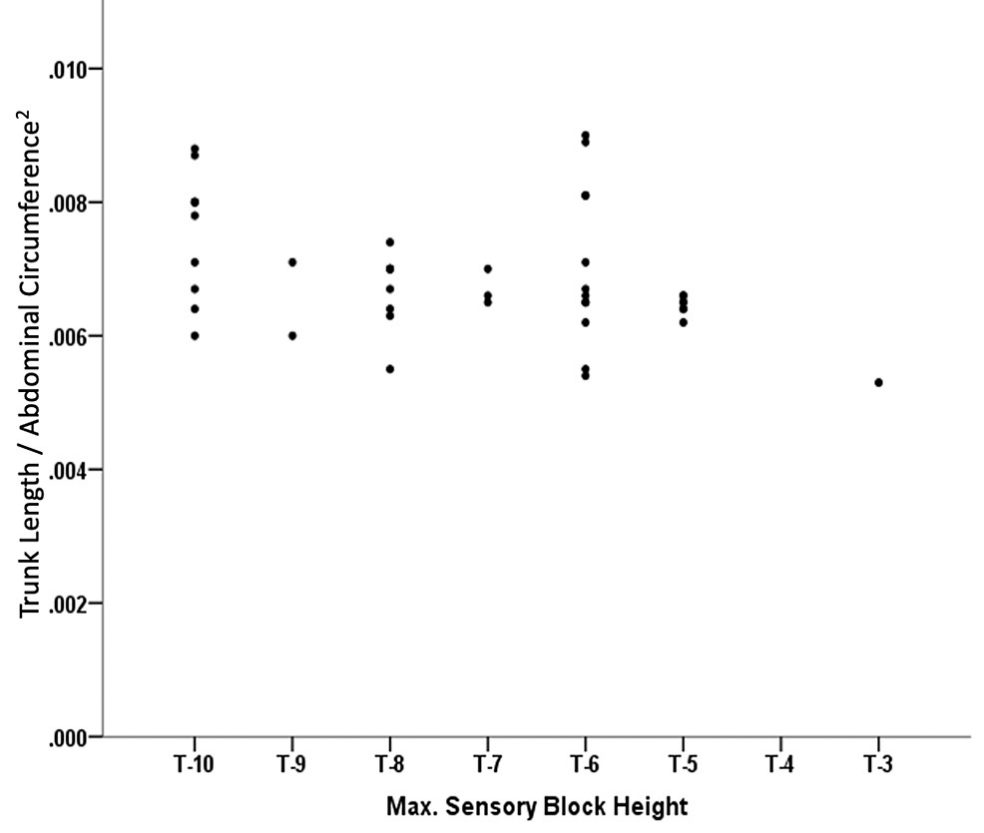
Relationship of Trunk Length/Abdominal Circumference2 (TL/AC2) to SABH

The co-morbidities of patients are shown in Figure 3. The mean TL of the patients was 63.02 +/- 5.71 (Mean +/- SD), and the TL/AC^2^ ranges from 0.005-0.009, as shown in Table 2. The lowest SABH observed was at the T-10 level, while the highest was at the T-3 level, which is evident from Figure 4.

**Table 2:** Measurements TL, AC and TL/(AC)2

Variables	Mean ± SD	95% Confidence Interval for Mean		Min-Max
		LowerBound	UpperBound	
Trunk length cm	63.02±5.71	61.26	64.77	50-70
Abdominal circumference cm	95.29±8.68	92.62	97.96	77-112
Trunk length (TL) / Abdominal circumference (AC)^2^	0.0067±0.00126	0.0063	0.0071	0.005-0.009^2^

The spearman rank correlation coefficient between maximal sensory block height and TL/AC^2^ was -0.284. Therefore, the ratio of trunk length and square of abdominal circumference TL/AC^2^ was significantly related to spinal anesthesia block height in geriatric patients undergoing TURP (p=0.015), as shown in Table 3.

**Table 3:** Spearman Rank Correlation Coefficient Between Trunk Length/Abdominal Circumference2 (TL/AC2) and SABH

Statistics	Trunk Length /Abdominal Circumference^2^ (TL/AC^2^) to SABH
n	43
Correlation Coefficient	-0.284
P-Value	0.015

Abdominal circumference was found to be significantly related to block height (p=0.036) (Table 4). BMI also showed a significant relationship with spinal anesthesia block height (p=0.02) (Table 5).

**Table 4:** Spearman Rank Correlation Coefficient Between Abdominal Circumference (AC) and SABH

Statistics	Abdominal Circumference and SABH
n	43
Correlation Coefficient	-0.321
P-Value	0.036

**Table 5:** Spearman Rank Correlation Coefficient Between BMI and SABH

Statistics	BMI and SABH
n	43
Correlation Coefficient	-0.346
P-Value	0.023

## Discussion

The study was conducted to observe the relationship between the ratio of trunk length and square of the abdominal circumference with SABH in geriatric patients undergoing TURP. The results revealed a statistically significant relationship between the ratio TL/AC^2^ and SABH, i.e., r = -0.284 and p = 0.015 (r = Spearman's rank correlation coefficient). This means that geriatric patients with shorter trunk lengths and larger abdominal circumferences would tend to have higher SABH when performed with a standardized clinical technique and intrathecal local anesthetic solution.

Lee et al. conducted a similar study on term parturients undergoing lower-section cesarean section and found comparable results. They concluded that there is a significant relationship between TL/AC^2^ and spinal anesthesia block height; the spearman rank correlation coefficient (r) was -0.45 with p-value=0.02 [8]. Our study has revealed a comparable and statistically significant relationship with (r) = -0.284 and p-value= 0.015. Our sample size was 43, compared to 29 in the study by Lee et al. [8].

Multiple studies have been conducted to investigate the association between SABH and different patient characteristics [7,25,26]. Zhou et al. studied the relationship between vertebral column length and SABH in patients undergoing lower limb orthopedic surgery and found a strong link between the parameters (r = -0.243 and p = 0.009) [26]. In 2017, Chang et al. reported an even stronger correlation (r = -0.711, p < 0.0001) between TL and SABH in a study on the term parturient [27]. Hartwell et al. also studied this phenomenon in terms of parturient and found a positive relationship (r = 0.38 and p = 0.006) [18].

In this study, we did not find a statistically significant relationship between TL and SABH (r = -0.028). This lack of correlation can be explained by the geriatric nature of our study population. We included patients above the age of 60, while Zhou et al. excluded patients over the age of 55. This viewpoint was also highlighted by Pargger et al.; they found no correlation between trunk length and peak sensory level in elderly age group patients [28]. Therefore, age-related degenerative changes may have possibly played a role.

Zhou et al. [26] also studied the relationship between abdominal girth and SABH and found a strong relationship between both variables (r = 0.821 and p < 0.0001). Chang et al. studied this relationship in the term parturient and found a similar correlation (r = 0.372, p<0.0001) [27]. In our study, the relationship was statistically significant (r = 0.321 and p = 0.036) but with a weaker statistical difference compared to those found by Zhou et al. and Chang et al.

The mechanism by which abdominal circumference influences spinal anesthesia block height might be related to the changes in intra-abdominal pressure in sitting and supine positions. Intra-abdominal pressure rises as the patient lies down following the induction of spinal anesthesia [29]. This rise in intra-abdominal pressure will be proportional to the abdominal circumference and could result in the transfer of local anesthetics to the higher spinal levels [30]. Another possible mechanism could be the shifting of soft tissues to intervertebral foramina, causing a decrease in CSF volume [30,31]. Engorgement of the epidural veins, as seen in the parturient, can cause dura to encroach on the subarachnoid membrane, which could be another possible mechanism [32,33]. However, Seyhan et al. [34] studied the correlation of intra-abdominal pressure with SABH and concluded that there is no relationship between intra-abdominal pressure and block height.

As the results were conflicting when TL or AC was studied as an individual parameter, Lee and co-researchers [8] conceptualized the three-dimensional nature of the body and its influences on the spinal canal. They proposed that it is the three-dimensional aspect of the body that leads to discrepancies in correlations with SABH when studied using one dimension alone, i.e., long axis (TL) or short axis (AC).

Thus, Lee et al. [8] combined both the parameters, TL and AC, to discover a link between the ratio of both the parameters and spinal anesthesia block height. The premise was that by taking both characteristics into account, the correlation would improve.

This hypothesis can be rationalized by looking at the three-dimensional nature of the body. If we draw the transection area of the abdomen as an ellipse or circle, the diameter of this circle will have the same trend as the abdominal circumference (AC). On the other hand, AC^2^ will represent the maximal transection area of the abdomen. Therefore, TL/AC^2^ is the ratio between the long axis and transection area of the abdomen. When considered individually, TL and AC have the opposite effect on the SABH. Their ratio has the potential to improve the correlation with SABH.

Lee et al. [8] identified a positive relationship between TL/AC^2^ and sensory block height in the parturient, while our study identified a similar relationship in the geriatric population. In our study, all the parameters, including spinal level (L3-L4), drug dose (0.5% hyperbaric bupivacaine 10 mg), and technique, were standardized for all the patients.

Limitations

It should be noted that our study has some limitations. The study was conducted on a geriatric population aged between 60 and 80 undergoing TURP with 0.5% hyperbaric bupivacaine spinal anesthesia. Therefore, the results could not be generalized to patients of all ages undergoing various surgeries under spinal anesthesia. As the study population was undergoing TURP in a lithotomy position, it could have influenced the block height [35]. The spinal level was identified through the intercristal line (i.e., Tuffier’s line) using the palpatory method. This method is not 100% accurate, and discrepancies could occur, as reported by Christopher and colleagues [36]. Similarly, the C-7 spinous process can be identified correctly in only 77.1% of the population by the palpatory method [23]. Successful identification of sacral hiatus through the palpatory method is reported in up to 75% literature [37-39]. These two factors can influence trunk length measurement.

## Conclusions

Adequate spinal anesthesia block height is necessary to avoid side effects or complications of spinal anesthesia and provide adequate surgical conditions. It is difficult to predict the spread of local anesthetics in subarachnoid space, and various methods are used to predict the block height. This observational study shows a significant correlation between spinal anesthesia block height and the ratio of trunk length and abdominal circumference, which coincides with the ratio of the long axis and transection area of the abdomen in geriatric patients. Therefore, a geriatric patient with a short trunk length and a large abdominal circumference might have a high spinal anesthesia sensory block height.

However, it would be unrealistic to consider a single factor as the sole determinant of spinal anesthesia block height, keeping in view the complexity of mechanisms of intrathecal drug spread. Clinicians should consider all the relevant factors while administering spinal anesthesia and not rely on a single factor.
